# Supplementary material for: Quantification of Death Risk in Relation to Sex, Pre-Existing Cardiovascular Diseases and Risk Factors in COVID-19 Patients: Let’s Take Stock and See Where We Are
Source: J Clin Med. 2020 Aug 19;9(9):2685. doi: 10.3390/jcm9092685 (PMC7564581; doi:10.3390/jcm9092685)
Supplement: Supplementary file 1 [file jcm-09-02685-s001.zip › Supplemental Material.docx]

**Figure Legend**

**Supplemental Figure 1.** Funnel plot of **A.** Sex. **B.** Age. **C.** Overall CVD (CVD+CAD) vs patients without CVD. **D.** Patients with CAD vs patients without CAD. **E.** Patients with CVD (and no CAD) vs patients without CVD-CAD. **F.** Patients with hypertension vs patients without hypertension. **G.** Patients with cerebrovascular diseases vs patients without cerebrovascular diseases. **H.** Patients with diabetes mellitus vs patients without with diabetes mellitus.
