# Supplementary figures and images for: Quantification of Death Risk in Relation to Sex, Pre-Existing Cardiovascular Diseases and Risk Factors in COVID-19 Patients: Let’s Take Stock and See Where We Are
Source: J Clin Med. 2020 Aug 19;9(9):2685. doi: 10.3390/jcm9092685 (PMC7564581; doi:10.3390/jcm9092685)

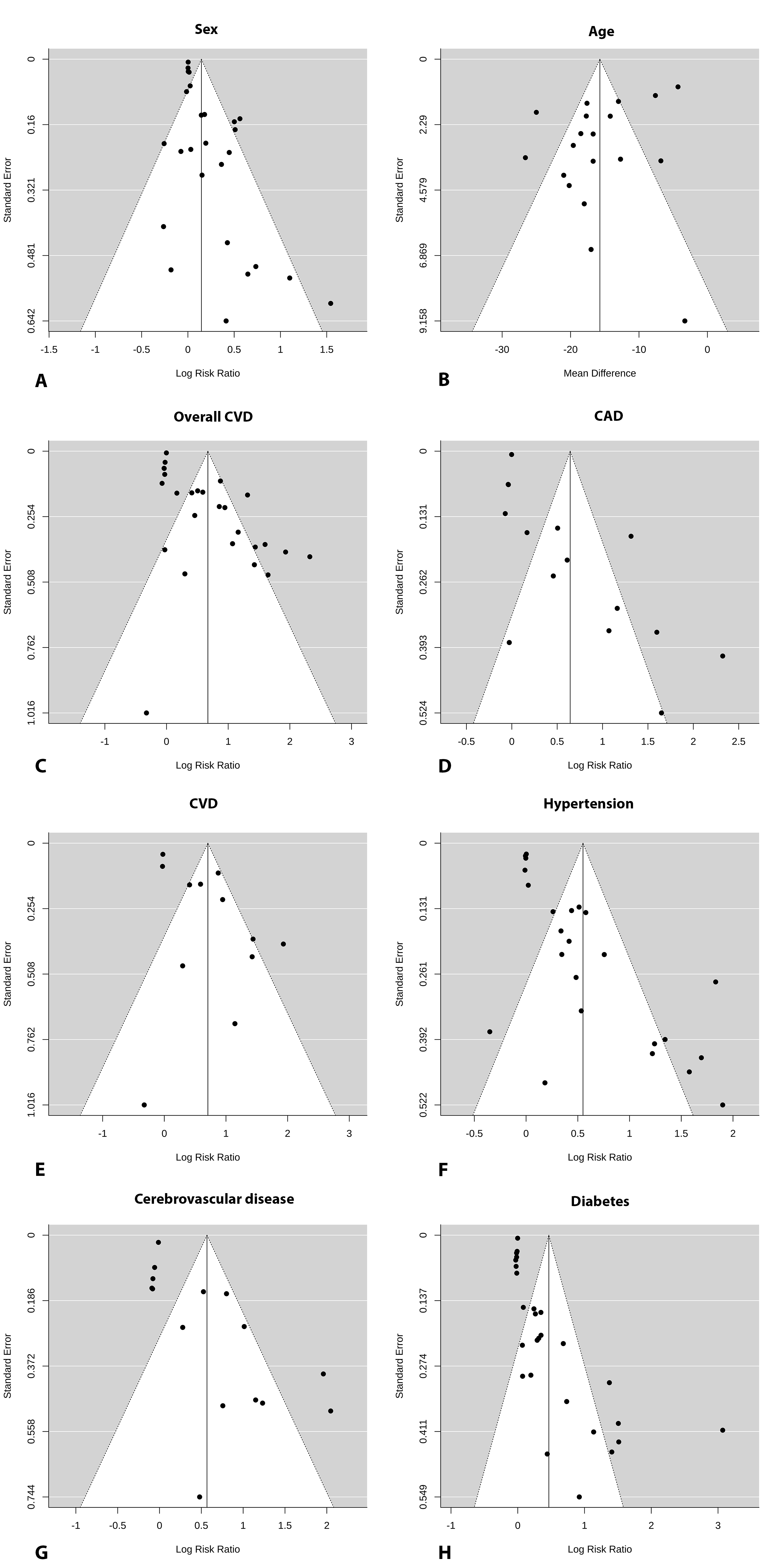

Supplement: Supplementary file 1 [file jcm-09-02685-s001.zip › Supplemental Figure 1.tif]
